# Research on optimal allocation of water and land resources in the Yiluo River Basin, China

**DOI:** 10.1016/j.isci.2024.111739

**Published:** 2025-01-04

**Authors:** Tianling Qin, Denghua Yan, Jun Hou, Xizhi Lv, Weizhi Li, Jianming Feng

**Affiliations:** 1 School of Resources and Environment, Anqing Normal University, Anqing 246133, China; 2 State Key Laboratory of Simulation and Regulation of Water Cycle in River Basin, China Institute of Water Resources and Hydropower Research, Beijing 100038, China; 3 Yellow River Institute of Hydraulic Research, Zhengzhou 450003, China

**Keywords:** Environmental science, Environmental management, Environmental policy, Natural resources, Environmental resource, Land use

## Abstract

The allocation of water and land resources (WLRs) has adhered to a system where they “serve as boundaries for each other,” posing challenges in satisfying the exacting management requirements for these resources in the contemporary era. In line with precise predictions of the future WLRs, the optimal allocation framework of WLRs was constructed to obtain the rational configuration scheme. The prediction results showed that the imbalance between supply and demand of WLRs will intensify in the Yiluo River Basin. After configuration, the water shortage, water consumption, pollutant discharge, and soil erosion will be mitigated, and the ecosystem service function will be enhanced during the research period. It was proposed to enhance the WLR quality by strengthening the supply capacity, raising the utilization efficiency, reducing pollution, and safeguarding the ecological function. The research results will provide theoretical and technical references for the meticulous management of WLRs.

## Highlights

• The future water and land resources under different SSP scenarios were predicted
• A multi-objective optimal allocation model of water and land resources was established
• The model was calculated by non-dominating genetic algorithm

## Graphical abstract

**Figure:**
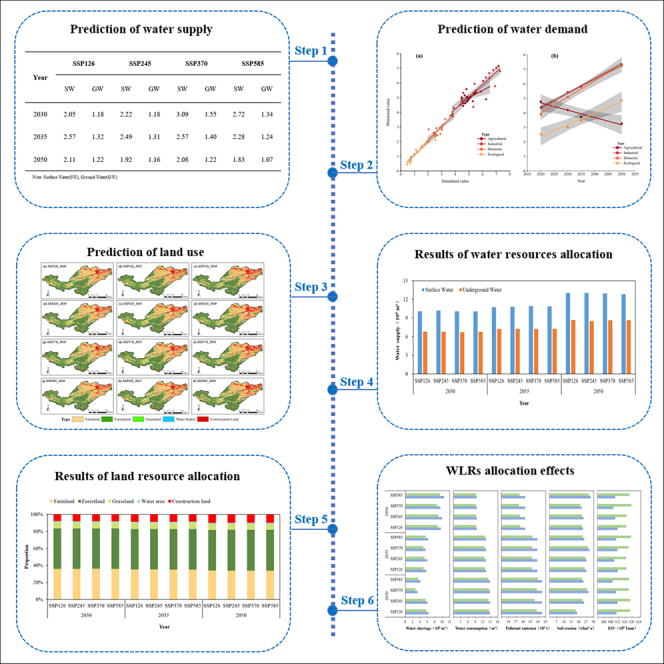

## Introduction

Irrational water and land resources (WLRs) exploitation was the key factor causing the inequality and inadequacy of socio-economic development, as well as the inducement of ecological environment disruption.^1^^,^^2^^,^^3^ Alongside rapid economic expansion and population swells, the WLRs demand increased dramatically.^4^^,^^5^ The excessive WLRs exploitation by various departments such as agricultural, industrial, domestic, and ecological has led to serious degradation of regional ecological environment.^6^^,^^7^ Meanwhile, in the context of climate warming, the global water cycle rate has been accelerated. The amount-composition-distribution of water resources has undergone profound changes, and the water resource flux such as natural river runoff has declined.^8^^,^^9^ The water availability and supply have diminished, following which the quota of water demand is increasing sharply, resulting in a prominent conflict between WLR supply and demand.^10^^,^^11^ We illustrate that it is a scientific issue to be solved urgently to carry out the research on the WLR joint regulation.

After decades of exploration by several generations of researchers, the theory and technology of water resource allocation and land resource allocation have been greatly developed.^12^^,^^13^ The water resource allocation objectives have been developed from the water quantity allocation during the “Sixth Five-Year Plan” to the joint allocation of water quantity-quality-ecology-efficiency currently in China.^14^^,^^15^ Pei et al.^16^ developed a water resource regulation model oriented toward the water quantity-quality-efficiency and applied in the Nanliu River basin. The land resource allocation objects have developed from the allocation of productive and domestic land to the territorial space optimization.^17^^,^^18^ However, numerous studies have found that there was a mutual-feed relationship between water resources and land resources with physical mechanism.^19^^,^^20^ Water resource conditions and its changes directly affect the economic and ecological functions of land resources. The allocation pattern of land resources affects the total amount, composition, and spatiotemporal distribution, as well as the effectiveness, controllability, and renewability of water resources.^21^^,^^22^

Over the past few years, some scholars have tried to carry out the WRL joint regulation. The allocation objects mainly oriented to a single land type such as farmland, construction land, or ecological land.^23^^,^^24^ Smout and Gorantiwar^25^ proposed an agricultural WRL allocation model and obtained the optimal allocation scheme in a medium-sized irrigation district in India. The allocation targets included utilization efficiency, environmental protection, ecological restoration, and carbon neutrality.^26^^,^^27^ He et al.^28^ evaluated the carbon sequestration capacity and carbon emission capacity of different crop planting modes during the water resource allocation and achieved regional carbon neutrality and conservation of water and energy. The allocation model included linear optimization, dynamic optimization, multi-criteria decision analysis, system dynamics, and other methods.^29^^,^^30^ Ren et al.^31^ noted a multi-objective programming framework and obtained the optimal irrigation and planting structure scheme in Wuwei City under various uncertain conditions.

However, the published research on WRL optimal allocation mainly focused on a single allocation goal or allocation object and ignored the mutual interaction mechanism of them.^32^^,^^33^ Our innovation was to integrate the future WLR prediction technologies (Table 1) to develop an allocation model of WLRs that incorporated their interaction relationships. The objectives of the model involved water quantity, water quality, and water-land use efficiency, while simultaneously accounting for constraints such as water resource balance, land resource balance, and water-land use red lines. Subsequently, a non-dominated sorting genetic algorithm (NSGA) was employed to propose the optimal regulation schemes and management measures for WLRs in the target area. Our study further developed the WLR optimal allocation technology and broke through the puzzle of water resource and land resource allocation that “acted as each other’s boundary.”

**Table 1:** Future surface and underground water availability under different SSP scenarios (billion m³)

Year	SSP126		SSP245		SSP370		SSP585	
	Surface water	Ground water	Surface water	Ground water	Surface water	Ground water	Surface water	Ground water
2030	2.05	1.18	2.22	1.18	3.09	1.55	2.72	1.34
2035	2.57	1.32	2.49	1.31	2.57	1.40	2.28	1.24
2050	2.11	1.22	1.92	1.16	2.08	1.22	1.83	1.07

**Figure 1:**
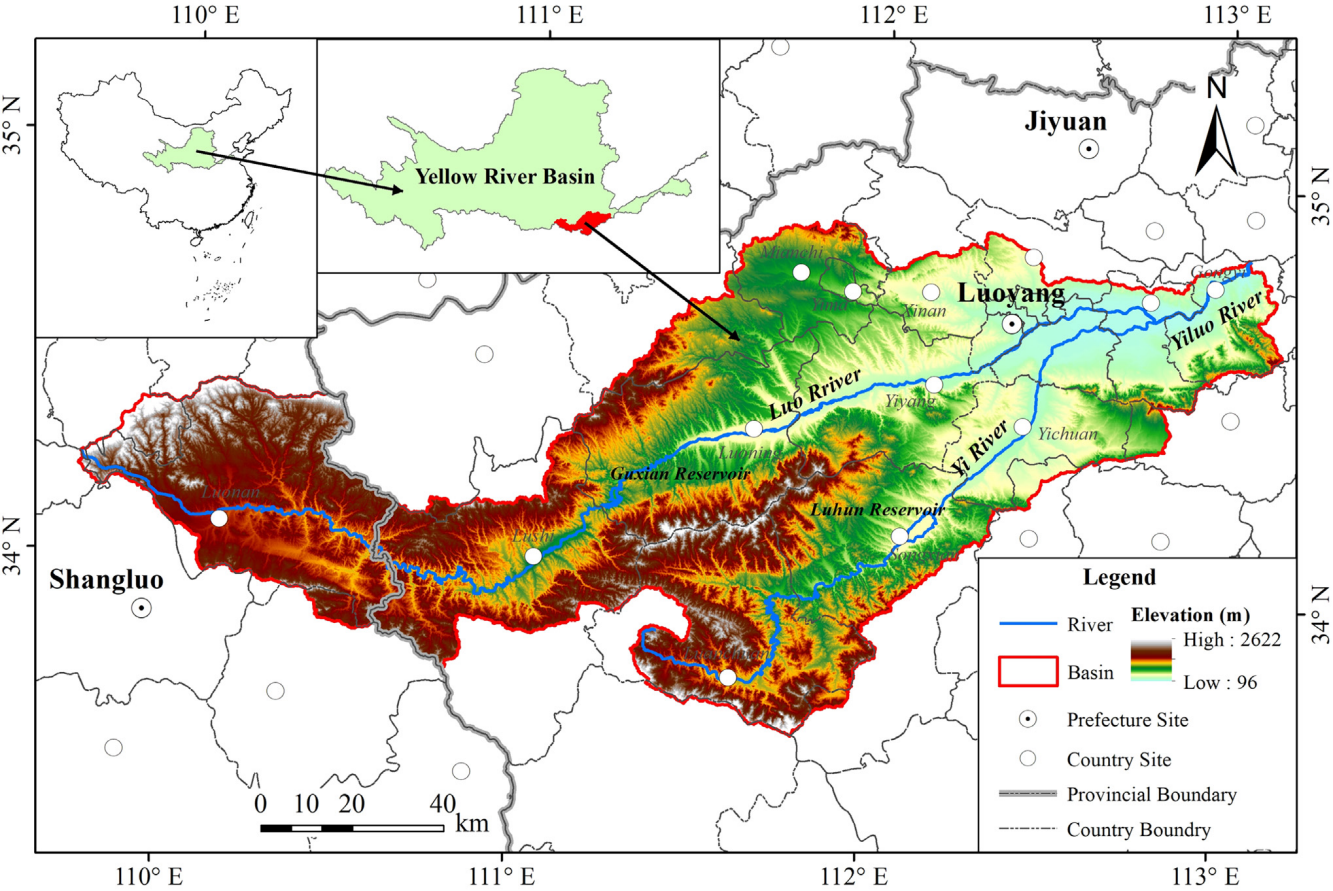
The watershed geographical location

**Figure 2:**
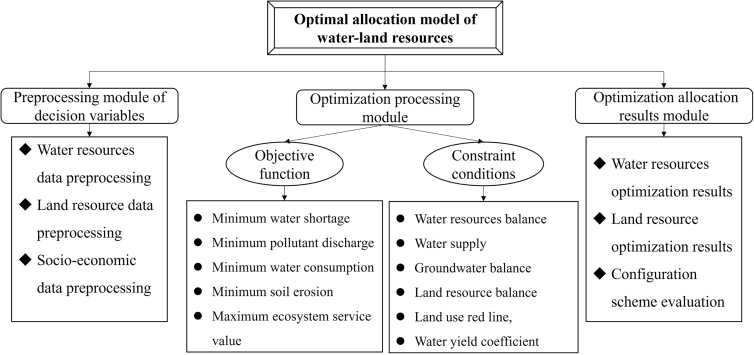
The structure of water-land resources optimal configuration model

The Yiluo River Basin is a tributary with an important strategic role in the lower reaches of the Yellow River Basin (Figure 1). With the rapid socio-economic development, productive and domestic water consumption increased, resulting in the water competition between industrial and agricultural sectors, urban and rural areas, and upstream and downstream areas. Simultaneously, frequent extreme events led to a downward trend in the availability and controllability of water resources, further expanding the imbalance between WLR supply and demand. The purposes of our study were: (1) to construct an optimal allocation model of WLRs (Figure 2) and (2) to obtain the optimal allocation scheme of WLRs under diverse shared socio-economic pathway (SSP126, SSP245, SSP370, and SSP585) scenarios in the future.

## Results

### Prediction and analysis of future WLRs

(1) Prediction of water supply

Future available water supply was predicted by the water and energy transfer process (WEP) model. The results showed that the model has a better simulation effect and can be used for further calculation in the Yiluo River Basin.^34^ The surface and underground water resources in 2030,2035, and 2050 under different SSPs were simulated (Table 2). The results indicated that the surface water resources range from 2.09 to 2.579 billion m³, and the groundwater resources range from 1.216 to 1.391 billion m³. Under different SSP scenarios, the surface and underground water supply will be the largest under SSP370 scenario and the smallest under SSP245 scenario from 2030 to 2050.

(2) Prediction of water demand

**Table 2:** Prediction methods of future water-land resources

Types	Methods	Explanation
Future climate model data	linear scaling method	precipitation, temperature, wind, radiation, humidity
Future water resources available	WEP model	availability of surface and underground water resources
Future land use	FLUS model	land use changes
Future water demand	trend extrapolation method	socio-economic indicators, water consumption per unit
	linear regression model	ecological water demand
	quota method	agricultural, industrial, and domestic water demand

**Figure 3:**
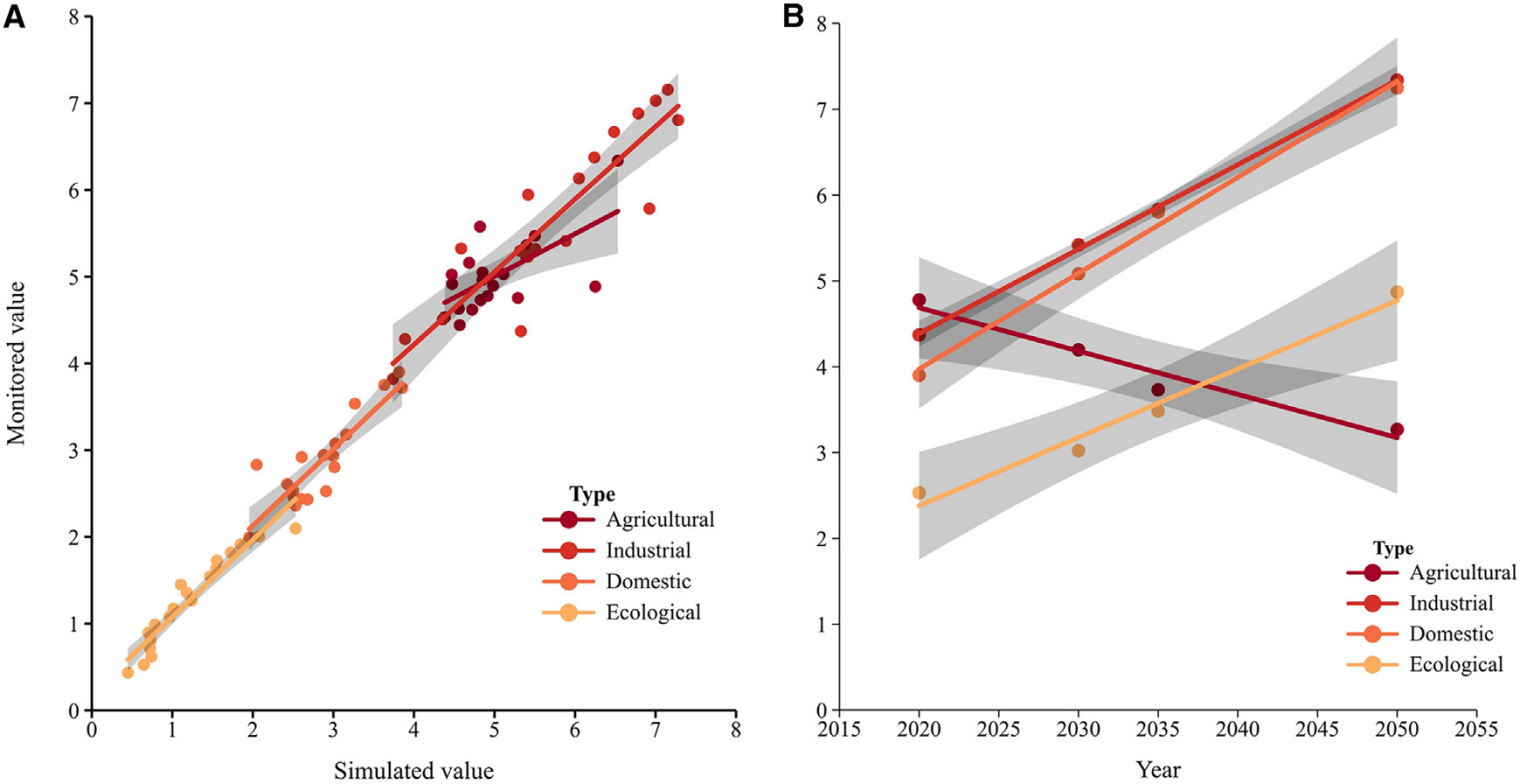
Simulation results of water demand (A) Simulation effects of different water demand sectors in 2001–2020. (B) Simulation results of different water demand sectors in 2030–2050.

On the strength of water demand prediction method, the water utilization for agricultural, industrial, domestic, and ecological sectors in the Yiluo River Basin was simulated from 2001 to 2020 (Figure 3). The simulation results showed that the correlation coefficient of water demand in each sector was 0.65–0.92, and the relative error was 0.56%–10% (Figure 3A). Illustrating that the simulation effect of water utilization of each sector is good, the method was further applied to predict the future water demand in 2030, 2035, and 2050 (Figure 3B). It was estimated that the water demand for agricultural, industrial, domestic, and ecological sectors will account for 31.35%, 18.88%, 30.58%, and 19.18% in 2030, 2035, and 2050, respectively. Compared with 2020, the water demand for domestic, industrial, and ecological sectors will be increased by 30.33%–85.86%, 23.99%–67.91%, and 19.41%–92.43%, respectively; the agricultural water demand will be reduced by 12.17%–31.59%.

(3) Prediction of land use

**Figure 4:**
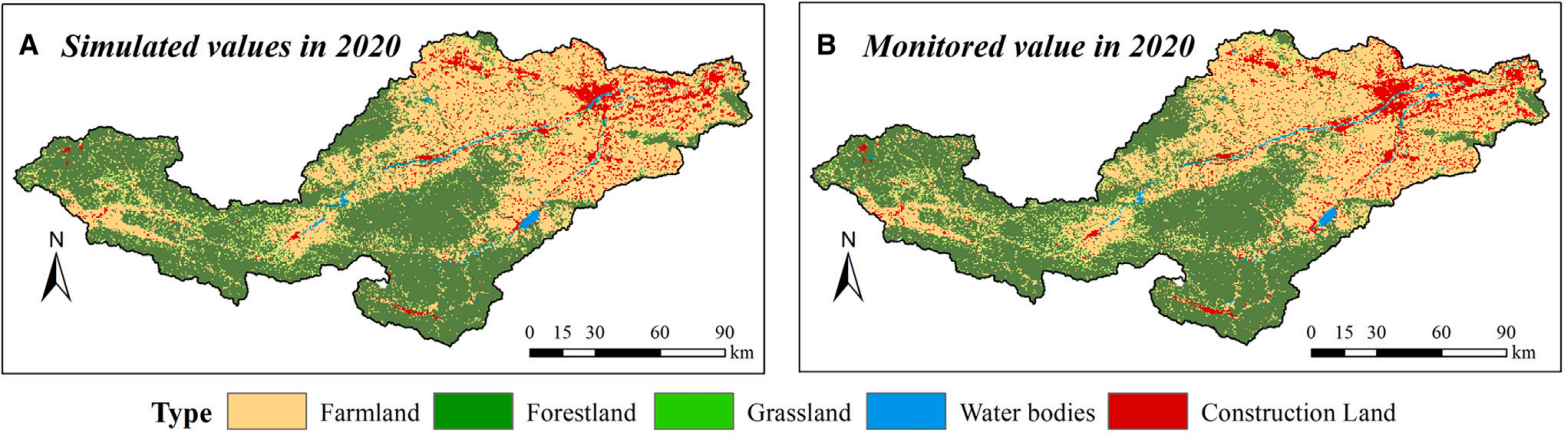
Simulation effects of land resources

**Figure 5:**
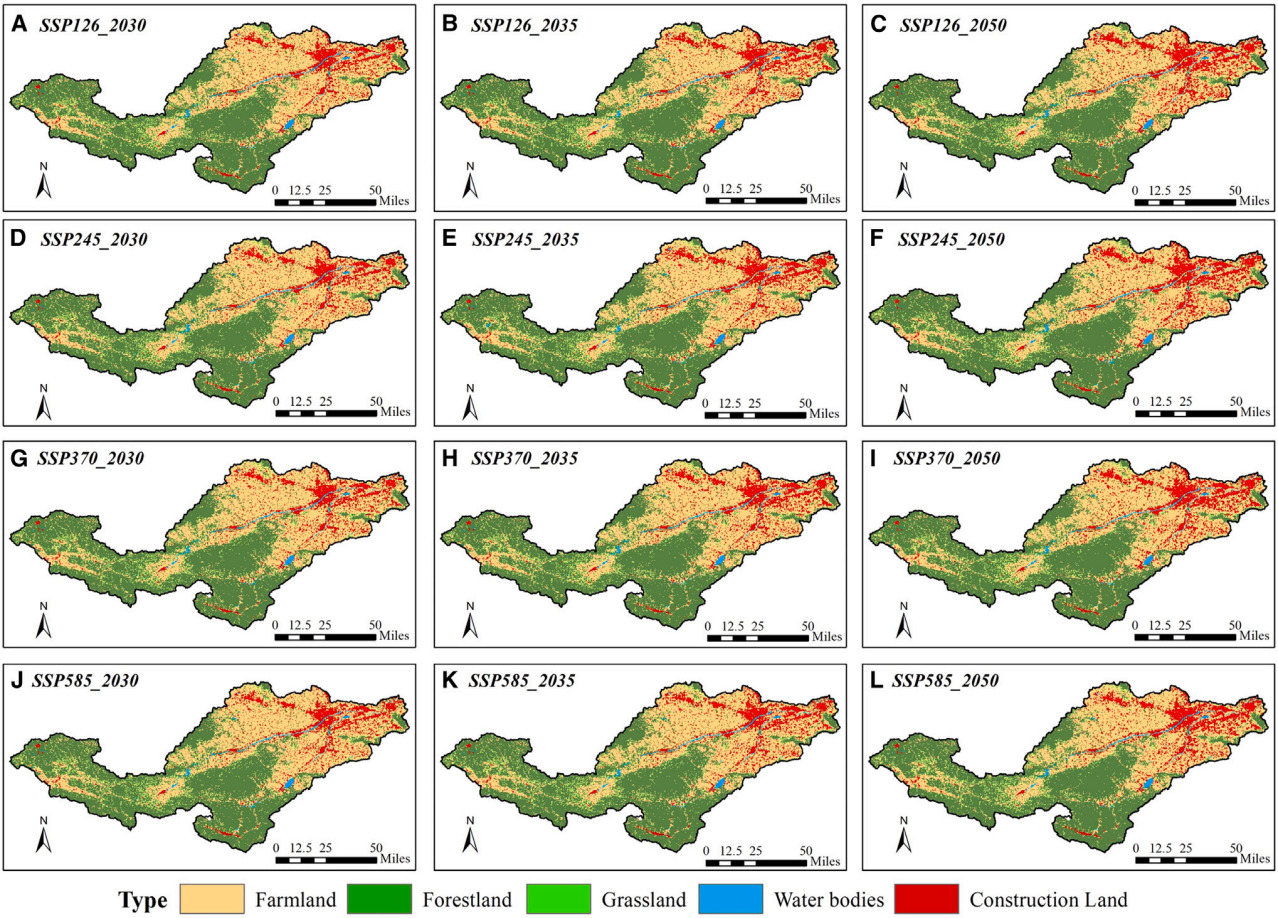
Land utilization distribution in 2030, 2035, and 2050 under different SSP scenarios

The future land use simulation (FLUS) model was applied to simulate the land utilization in 2020; the results manifested that the Kappa coefficient was 0.87, signifying that the model could better simulate the land utilization in the Yiluo River Basin (Figures 4A and 4B). Then the land resources in 2030,2035, and 2050 under different SSP scenarios were simulated (Figures 5A–5L). Compared with the current year, the cultivated land area will continue to decrease by 3.39%–8.92% from 2030 to 2050. The forestland and impervious area will increase by 0.69%–1.48% and 16.7%–43.42%, respectively, while the grassland area will be the largest under SSP126 scenario and the smallest under SSP370 scenario, decreased by 2.79%–5.95% and 3.43%–6.74%, respectively. The water area will be the largest under SSP370 scenario and the smallest under SSP126 scenario, increased by 1.44%–2.57% and 0.33%–1.17%, respectively.

### WLR optimal configuration

(1) Results of water resource allocation

**Figure 6:**
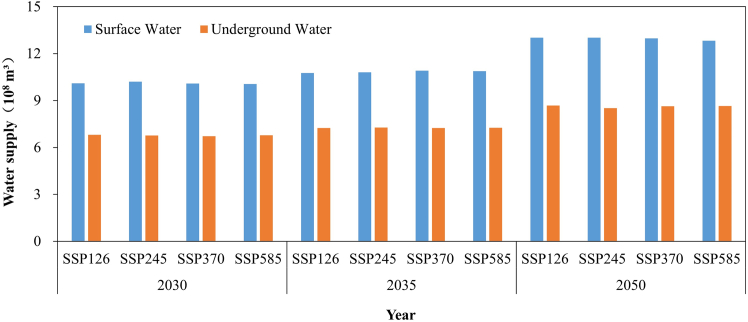
Future surface and underground water resources supply under different SSP scenarios in 2030, 2035, and 2050 after configuration

On the strength of the NSGA-II optimization algorithm, the supply quantities of surface and underground water resources in the Yiluo River Basin under different SSP scenarios in 2030, 2035, and 2050 after configuration were obtained (Figure 6). The results showed that the water supply quantity of surface and underground water resources will be 11.25–11.35 10^8^ m³ and 7.52–7.58 10^8^ m³, respectively. Compared with earlier configuration, it will be reduced by 5.32%–6.06% and 5.15%–5.83% under different SSP scenarios, of which the water supply of surface water resources is the highest under SSP245 scenario and the least under SSP580 scenario. The water supply of groundwater resources will be the largest under SSP126 scenario and the lowest under SSP245 scenario.

(2) Results of land resource allocation

**Figure 7:**
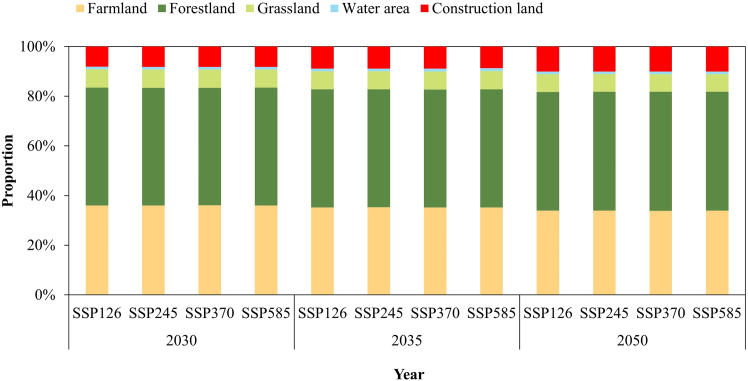
Future land utilization proportion under different SSP scenarios in 2030, 2035, and 2050 after configuration

Based on the NSGA-II optimization algorithm, future land utilization proportion under different SSP scenarios in 2030, 2035, and 2050 after configuration was obtained (Figure 7). The results showed that the area of forestland, grassland, and water bodies will mainly present a growth trend, while farmland and construction land will present a downtrend under different SSP scenarios after configuration. Compared with pre-allocation, the area of forestland, grassland, and water bodies will be increased by 0.56%–1.22%, 0.37%–1.02%, and 0.53%–0.64%, respectively, while the cultivated land and construction land will be decreased by 0.2%–0.54% and 3.97%–4.91%, respectively.

(3) WLR allocation effects

**Figure 8:**
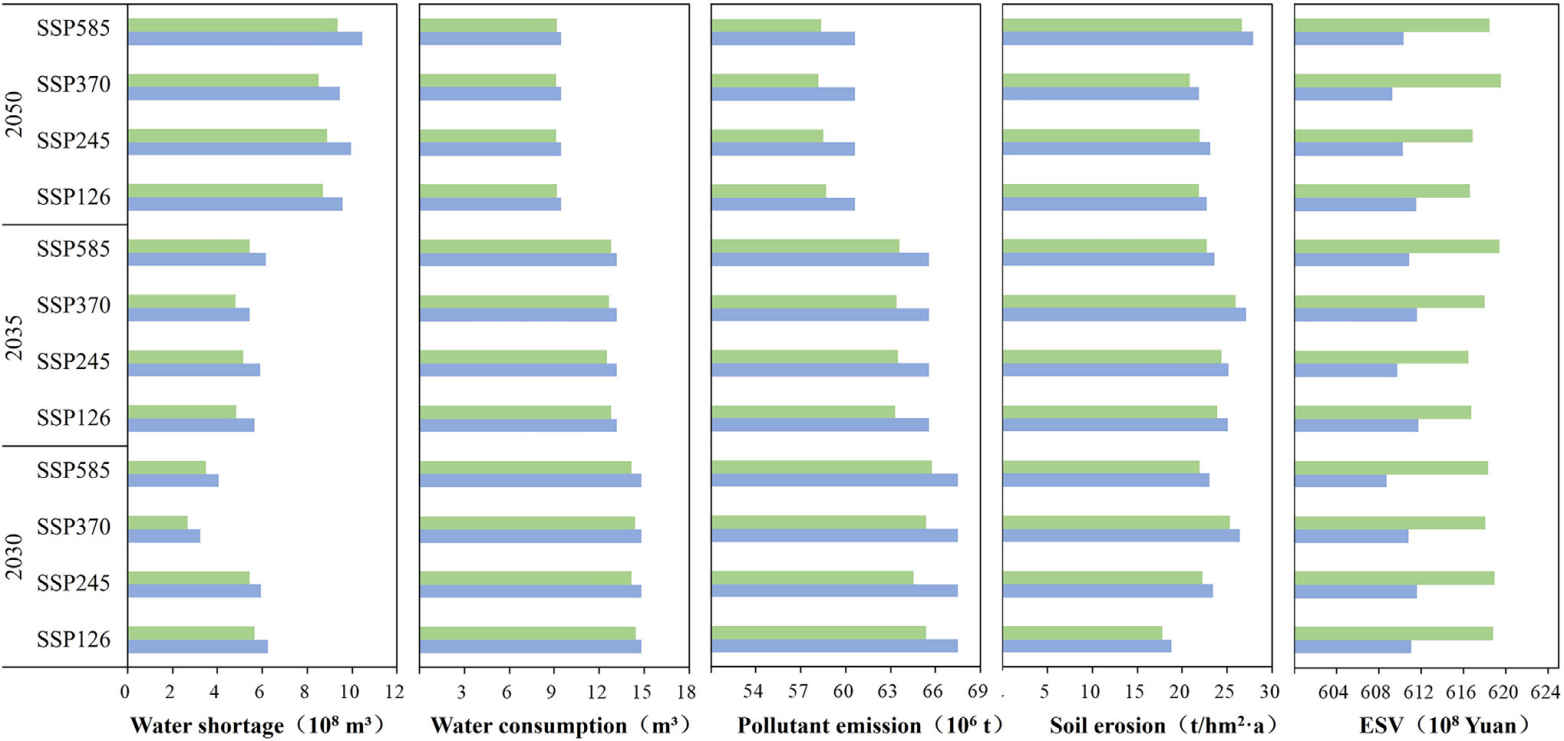
Effect of water-land resources allocation

The WLRs allocation effects in Yiluo River Basin in 2030, 2035, and 2050 under the different scenarios were further analyzed (Figure 8). The water shortage, water consumption, pollutant discharge, soil erosion, and ecosystem service value of the basin will be 532–649 million m³,11.93–12.14 m^3^, 6,216.83–6,256.74 10^4^ tons, 21.17–24.03 $t/\left({\text{hm}}^{2}\cdot a\right)$, and 61.74–61.87 billion yuan, respectively. Compared with pre-allocation, the preceding four indicators will be decreased by 10.62%–13.21%, 2.84%–4.4%, 3.12%–3.7%, and 4.29%–4.66%, respectively. The last-mentioned indicator will be increased by 0.97%–1.43%.

## Discussion

After allocation, the WLR quality and efficiency in the Yiluo River Basin will be improved markedly, while some engineering and non-engineering measures are still needed to protect the watershed WLRs. Specific measures include strengthening the supply capacity, raising the utilization efficiency, reducing pollution, and safeguarding the ecological function of WLRs.

(1) Strengthening the supply capacity of WLRs. Due to the improvement of water use efficiency, as well as the change of water and land use types, the water shortage in the basin under different SSP scenarios reduced by 10.62%–13.21%. However, there was still a large water shortage gap in the lower watershed, which was basically featured as resource-oriented water shortage. In the next planning, it was necessary to further improve the water resources regulation technique to enhance the capacity of watershed water supply security.^35^^,^^36^^,^^37^ Simultaneously, the development and utilization of unconventional water resources such as rainwater and renovated water resources must be strengthened.^38^^,^^39^ In the midstream and upstream of the watershed, water resources were relatively abundant, but engineering-oriented water shortage often occurred. The precipitation was concentrated in flood season, exhibiting the obvious feature of seasonal water shortage. It was recommended to build more reservoirs, dams, or some other water storage projects in the most cost-effective and feasible locations and minimize its environmental and social impacts, improving the regulation capacity of water resource, as well as the ability of drought and flood regulation.
(2) Raising the utilization efficiency of WLRs. By implementing water quota controls, the water consumption reduced by 2.84%–4.4%, and there was still a big gap compared with the developed cities and regions, mainly due to the high input of WLRs and the low industrial output value and grain output.^40^ It was proposed that water saving and land saving should be taken as the premise for the development, utilization, protection, allocation, and scheduling of WLRs in the future, and the mode of water-land use should be further transformed into economizing and intensive.^41^^,^^42^ Confronting water resource insufficiency, it was necessary to comprehensively promote agricultural water conservation and efficiency enhancement, industrial water conservation, and emissions mitigation, as well as domestic water savings and leakage reduction, to reduce unnecessary water depletion.^43^ Regarding the industrial-oriented and domestic-oriented land, it was necessary to enhance the efficiency of water and land utilization via technological advancements and industrial restructuring. For the ecological-oriented land, especially the artificial ecosystem, it was essential to develop fine water-saving irrigation technology. In the meantime, negative effects such as decreased water yield and increased local water consumption caused by excessive ecological construction should be avoided.
(3) Reducing pollution. Due to the reduction in water consumption, as well as the enhancement of sewage discharge management and control, the pollutant discharge reduced by 3.12%–3.7%. Nevertheless, the point source pollution and the diffuse pollution were still serious in some local areas, especially in the midstream and downstream areas where human activities are more concentrated. It was suggested that water pollution treatment should be based on the perspective of water circulation to curb or reduce pollutants entering water bodies.^44^ Regarding the serious point source pollution areas, it was recommended to force the enterprises to carry out green technology innovation through water quality target management, reduce pollution emission, and strike a harmonious equilibrium between economic prosperity and environmental conservation, providing opportunities for the development of some green environmental protection and energy-saving industries and becoming a new driving force for regional economy. Considering the agricultural non-point source pollution, it was suggested to minimize the utilization of chemical fertilizers and pesticides and reduce pollutant emissions from the source, simultaneously, through the double control of multi-level water production and water consumption, to amplify the water fluidity, nurture the ecological balance, and elevate the water body pollution resilience.
(4) Safeguarding the ecological function. Due to the increasement of ecological land such as forestland and water bodies, soil erosion reduced by 4.29%–4.66%, and the ecosystem service value improved by 0.97%–1.43%. However, soil erosion remained a serious problem in the midstream, and the ecosystem service function was relatively poor in the downstream. To minimize and avoid the disturbance of social and economic development to the natural ecosystem functions, it was suggested to strengthen the following two works. First, enhance the natural ecological functions of social-economic system, such as optimizing the function of urban green space and deepening the construction of ecological irrigation district. Second, implement the ecosystem “function compensation” mechanism. For example, the scale and quality of forested area and grass-covered area in the vicinity of cities and arable land should be further improved, and the ecological functions weakened by social and economic activities should be enhanced.^45^

### Conclusion

Our research centered around the efficient allocation of WLRs in the Yiluo River Basin. After configuration, due to the improvement of water use efficiency, as well as the change of water and land use types, the water shortage will be reduced by 10.62%–13.21%, and the availability of surface waters and groundwaters will be decreased by 5.32%–6.06% and 5.15%–5.83% under different SSP scenarios from 2030 to 2050, respectively. With the structure and scale change of land resource, especially the increasement of ecological land and the decrease of production land, pollutant discharge and soil erosion in the Yiluo River Basin will be reduced by 3.12%–3.7% and 4.29%–4.66%, respectively, and the ecosystem service function will be improved by 0.97%–1.43%. Although the quality and utilization efficiency of WLRs in the Yiluo River Basin have been significantly improved after allocation, we proposed that the quality of WLRs should be improved from strengthening the supply capacity, raising the utilization efficiency, reducing pollution, and safeguarding the ecological function.

### Limitations of the study

The linear scaling method was employed to modify the future climate model data in our research, being widely used in this field and proven to be an efficient approach. Recently, machine learning has gained extensive application in future climate predictions. Recognizing the potential of machine learning in related domains, we intend to integrate the method to further enrich and deepen our research findings in the future. Furthermore, given that the objective functions in our research were relatively simple, the NSGA-Ⅱ was chosen to procure the solution set. In future research endeavors, additional complex objectives such as carbon emissions and extreme water resource risk will be established. It would be worthwhile to introduce the NSGA-Ⅲ or other more advanced algorithms into this research domain to further explore and optimize solutions for multi-objective problems.

## Resource availability

### Lead contact

Further information and requests for resources will be fulfilled by the lead contact, Jun Hou(172309@aqnu.edu.cn).

### Materials availability

This study did not generate new unique materials.

### Data and code availability

• Data: the workable data mainly included the hydro-meteorological information, the land surface information, the water resources data, the socio-economic data, and the future climate model data. The datasets are listed in the key resources table.
• Code: this paper does not report original code.
• Any additional information required to reanalyze the data reported in this paper is available from the lead contact upon request.

## Acknowledgments

This research was supported by the National Science Fund Project (grant no. 52130907, 52409002, and 52342905), the Major Science and Technology Project of the 10.13039/501100004177Ministry of Water Resources of the People's Republic of China (SKS-2022033), and the Five Major Excellent Talent Programs of IWHR (WR0199A012021).

## Author contributions

T.Q.: conceptualization and writing – review and editing. D.Y.: supervision and validation. J.H.: methodology and writing – original draft. X.L.: validation and visualization. W.L.: software and data curation. J.F.: software and data curation.

## Declaration of interests

The authors declare no competing interests.

## STAR★Methods

### Key resources table

**Table:** 

REAGENT or RESOURCE	SOURCE	IDENTIFIER
**Deposited data**		

Precipitation, Temperature, Sunshine duration, Relative humidity, Wind velocity	National Meteorological Science Data Center	http://data.cma.cn
Natural runoff	Yellow River Water Conservancy Commission of the Ministry of Water Resources	http://www.yrcc.gov.cn
DEM, Slope, Aspect	Geospatial Data Cloud	http://www.gscloud.cn
Land use	GlobeLand30	https://globeland30.org
Soil	National Cryosphere Desert Data Center	http://www.geodata.cn
Water resources, water consumption	The second National Water Resources Survey and Evaluation;Water Resources Bulletin	https://slt.henan.gov.cn/bmzl/szygl/szygb
Soil moisture	United States Geological Survey	https://lpdaac.usgs.gov
Soil erosion	Yellow River sediment bulletin	http://www.yrcc.gov.cn/gzfw/nsgb
GDP, Population,	Statistical Yearbook	https://tjj.henan.gov.cn/tjfw/tjcbw/tjnj
Precipitation, Temperature, Wind velocity, Radiation, Relative humidity	Coupled Model Intercomparison Project Phase 6	https://esgf-node.llnl.gov/projects/cmip6

**Software and algorithms**		

Origin 2021	OriginLab	https://www.originlab.com
MATLAB	MathWorks	https://www.mathworks.com
Microsoft EXCEL	Microsoft	https://www.microsoft.com
ArcGIS	ESRI	https://www.arcgis.com

### Method details

#### Study area

The Yiluo River basin is a typical third-level water resources region in the Yellow River Basin (Figure 1). The river is a "twin river", mainly composing of the master stream Luo River and the tributary Yi River. The two rivers meet in Guxian township, then flow into the Yellow River in Shendi village. The basin spans an area of 18.9 thousand km^2^, with a greatest length of 319.8 km in the east-west direction, and an extreme breadth of 138.7 km in the north-south direction. The watershed topography is dominated by mountains, hills, and plains. Of which the mountainous terrains are largely distributed in the western part, with an elevation of 1200-2622m, being the major water conservation area. The hills terrains are mostly located in the center and the north, with an elevation between 800-1200m, being the main soil erosion areas. The plains terrains are primarily located in the valley plain area of the downstream, being the gathering area of anthropogenic activities, and the eco-environment has been seriously damaged. The Yiluo River Basin is categorized as a continental monsoon climate zone, characterized by high temperature and abundant rainfall in summer, and low temperature and little rainfall in winter. The average annual precipitation is 680.88mm with large interannual fluctuations, and the average annual temperature is 12.63°C. Affected by climate change, the precipitation has shown a declining trend, while the temperature has exhibited a significant increase trend in recent years.

#### Prediction methods of future WLRs

We adopted a series of methods to predict and analyze the future WLRs (Table 1). With 2020 as the current year, the short-period planning to 2030, the medium- period planning to 2035, and the long- period planning to 2050 (which were consistent with planning periods mentioned in the High-quality development of the Yellow River). Simultaneously, to reduce the uncertainties of future climate model projections, the future WLRs under SSP126, SSP245, SSP370 and SSP585 were analyzed.^20^ Of which the future climate model data were modified by linear scaling method.^46^^,^^47^ Future water available resources were simulated by WEP model.^48^^,^^49^ Future land use was simulated by the Future land use simulation (FLUS) model.^50^^,^^51^ Future water demand was estimated by the trend extrapolation model, the linear regression model, and the quota methods.^52^^,^^53^

#### Optimal allocation model

The WLRs optimal allocation model included the preprocessing module of decision variables, the optimization processing module, and the optimization allocation results module (Figure 2). Among them, the optimization processing module was the core of the model, involving the allocation objectives and constraint conditions. The objective function included minimum water shortage, minimum pollutant discharge, minimum water consumption, minimum soil erosion, and maximum ecosystem service value. The constraint conditions included the water resources balance, water supply, groundwater balance, land resource balance, land use red line, and water yield coefficient.

Objective function of WLRs optimal configuration model included the function of minimum water shortage ($f_{1}\left(\vec{X}\right)$), minimum pollutant discharge ($f_{2}\left(\vec{X}\right)$), minimum water consumption ($f_{3}\left(\vec{X}\right)$), minimum soil erosion ($f_{4}\left(\vec{X}\right)$), and maximum ecosystem service value ($f_{5}\left(\vec{X}\right)$). The details were as follows:

(Equation 1) $$\begin{array}{c} f_{1}\left(\vec{X}\right)=\sum_{i=1}^{n}\sum_{k=1}^{p}\left[\sum_{j=1}^{m}\left({WR}_{ijk}\times {LR}_{ij}\right)-{WS}_{ik}\right] \end{array}$$

(Equation 2) $$\begin{array}{c} f_{2}\left(\vec{X}\right)=\frac{1}{n}\sum_{i=1}^{n}\frac{\sum_{k=1}^{p}\sum_{j=1}^{m}{WR}_{ijk}\times {LR}_{ij}}{{GDP}_{i}} \end{array}$$

(Equation 3) $$\begin{array}{c} f_{3}\left(\vec{X}\right)=\sum_{i=1}^{n}\sum_{j=1}^{m}e_{ij}\times c_{ij}\times {LR}_{ij}\times \sum_{k=1}^{P}{WR}_{ijk} \end{array}$$

(Equation 4) $$\begin{array}{c} f_{4}\left(\vec{X}\right)=\sum_{i=1}^{n}\frac{\sum_{j=1}^{m}{SE}_{ij}\times {LR}_{ij}}{\sum_{j=1}^{m}{LR}_{ij}} \end{array}$$

(Equation 5) $$\begin{array}{c} f_{5}\left(\vec{X}\right)=\sum_{i=1}^{n}\frac{\sum_{j=1}^{m}{ESV}_{ij}\times {LR}_{ij}}{\sum_{j=1}^{m}{LR}_{ij}} \end{array}$$

where: *n*, *m*, and *p* represent the number of configuration unit, land utilization category and water resource category, respectively. ${WR}_{ijk}$ represents the water demand of land utilization type *j* per unit area for type *k* water resource in the configuration unit *i*. ${LR}_{ij}$ represents the proportion of land use type *j* in the configuration unit *i*. ${WS}_{ik}$ indicates the available water supply of class *k* water resource in the configuration unit *i*. ${GDP}_{i}$ represents the water consumption in the allocation unit *i*. $e_{ij}$ and $c_{ij}$ represent the pollutant concentration and pollutant emission coefficient, respectively. ${SE}_{ij}$ represents the soil erosion amount of land utilization type *j* in the configuration unit *i*. ${ESV}_{ij}$ represents the ecosystem service value of land utilization type *j* in the configuration unit *i*.

The constraint conditions of WLRs optimal allocation model included: gross amount of water resources balance, water supply, groundwater balance, land resource balance, land use red line, and water yield coefficient. The details were as follows:

(Equation 6) $$\begin{array}{c} \sum_{i=1}^{n}\sum_{k=1}^{p}{WS}_{ik}\leq \sum_{k=1}^{p}{TW}_{k} \end{array}$$

(Equation 7) $$\begin{array}{c} \min\left({WQ}_{j}\right)\leq {WS}_{ij}\leq \max\left({WQ}_{j}\right) \end{array}$$

(Equation 8) $$\begin{array}{c} {GWS}_{i}\leq {GWR}_{i} \end{array}$$

(Equation 9) $$\begin{array}{c} \sum_{i}^{n}\sum_{j}^{m}{LR}_{ij}=\sum_{j}^{m}A_{j} \end{array}$$

(Equation 10) $$\begin{array}{c} {\min\left({LR}_{ij}\right)\leq LR}_{ij}\leq \max\left({LR}_{ij}\right) \end{array}$$

(Equation 11) $$\begin{array}{c} {\bar{{WY}_{dry}}\leq WY}_{post}\leq \bar{{WY}_{wet}} \end{array}$$

where: ${TW}_{k}$ represents the water resources type *k* of total water resources quantity. ${WS}_{ij}$ represents the water supply of the land utilization type *j* in the configuration unit *i*. $\min\;\left({WQ}_{j}\right)$ and $\max\left({\text{WQ}}_{j}\right)$ represent the minimum water supply guarantee rate and maximum water supply quota of the land use type *j*, respectively. ${GWS}_{i}$ and ${GWR}_{i}$ represent the groundwater supply and groundwater replenishment in the allocation unit *i*, respectively. $A_{j}$ is the total area of land utilization type *j*, $\min\;\left({LR}_{ij}\right)$ and $\max\;\left({LR}_{ij}\right)$ represent the land use red line and the upper boundary of the land use type *j* in the configuration unit *i*, respectively. ${WY}_{post}$ represents the water yield coefficient after allocation. $\bar{{WY}_{dry}}$ and $\bar{{WY}_{wet}}$ represent the annual water yield coefficient in dry years and rainy years in historical period.

#### Optimization algorithm

The WLRs optimal allocation was a typical multiple target optimization process. Which involved multiple objectives and constraint conditions, needing to weigh the pros and con of different objectives, then proposed the optimal configuration scheme. The Elitist Non-Dominated Sorting Genetic Algorithm (NSGA-II) was adopted to realize the multi-objective optimization of WLRs allocation.^54^^,^^55^ NSGA-II was a multi-objective evolutionary algorithm on the strength of dominance relation, retaining the excellent individuals and the superiority of the group in the population better. By introducing the fast non-dominant algorithm and the elite strategy, the computational efficiency and accuracy of the algorithm were improved. Simultaneously, the introduced crowding degree operator solved the defect of artificially specifying the shared parameters, and ensured the superiority of the population.

During the process of model optimization calculation, the variation limits and constraint conditions for WLRs were closely aligned with their prediction results. Specifically, the water supply was predicted by the WEP model, encompassing both the available surface water and groundwater. The scope of water demand was determined by the water consumption patterns within the allocation unit. The land use change was predicted by the FLUS model. Based on the land use change patterns and allocation targets within the allocation unit, specific ranges were set for each land use type. The land use area and the red lines for various land use types within the allocation unit were served as the constraints for land resources. In terms of the water demand of various land use types, agricultural water was associated with cultivated land, industrial and domestic water were linked to construction land, ecological water was matched with forestland and grassland, while water bodies were not involved in the water allocation process. In addition, water pollution mainly originated from cultivated land and construction land, with agricultural water withdrawal, industrial and domestic wastewater discharge coefficients, and pollutant concentrations were used as pollutant constraints.

### Quantification and statistical analysis

All the datasets are listed in the key resources table, the methods are showed in the method details.
